# With a Garrison Battalion to Africa

**Published:** 1919-10-11

**Authors:** 


### WITH A GARRISON BATTALION TO AFRICA.

Our first sight of land was the hill at Freetown, Sierra Leone. A fine harbour, abounding with interest, awaited us. Everywhere one saw scores of natives lying in dug-out canoes with a foot over each side and a fishing-line on each big toe; dhows, or sailing boats, full of more or less naked blacks, apparently out just for pleasure. The town, with tiers of low white, red-roofed houses amongst coconut and betelnut palms, made one most eager to explore it at close quarters.

Boys of all sizes, from babies to six-footers, crowded round the landing-stage, offering to be our guides, and we selected a little fellow of about seven wearing just a loin-cloth and an old Tommy's cap. He proved to be very efficient, and spoke remarkably good English. But as to the rounds he took us, what had been beautiful to the eye did not thus appeal to the olfactory senses!

But in spite of the misgivings produced, during the stay at Sierra Leone we sent ashore to hospital only three mildly pyrexial cases, and generally the health of the men was very good. The C.O. insisted on every fit soldier on board doing at least half-an-hour's physical drill daily under expert instructors, and this undoubtedly helped to keep down the sick parades.

### Cape Town.

The later journey to Cape Town was medically uneventful, except for slowing the convoy on two or three occasions for a burial at sea, but in our own ship we had only one death and that from C.S.M. It was a sporadic case with very delayed symptoms and its true nature hard to realise at the outset. A *post-mortem*, however, showed typical meningitis, and the meningococcus was readily demonstrated. Possibly he was a carrier and the infection in reality secondary to lowered resistance due to heat exhaustion, several minor cases of which occurred.

To every traveller Cape Town has a strong appeal, and the shortness of the stay there caused many regrets, but our wishes were nevertheless to be fulfilled in the least-expected way. Next morning in glorious weather, sailing independently, having parted from the rest of the convoy, and happy in having come safely through the danger zone, we no longer looked upon our lifebelts as parts of ourselves, although regulations made us carry them about. Particularly songful were the men, with concertinas and mouthorgans setting the pace and tune, and at dinner, with these good spirits reflected in the officers, the meal promised to be a particularly lively one.

### Mines!

It was but half through when, without warning, a tremendous explosion shook the ship from stem to stern. Plates and glasses smashed everywhere, and for a moment turmoil prevailed. Many troops had already turned in and the water, crashing through the awnings above their hammocks, produced chaos indescribable. Gear of all kinds was scattered broadcast between-decks; men struggled to free themselves and each other. Noise and confusion was everywhere, but somehow—it is incredible how these things happen—within minutes the men were numbering off at their stations to abandon ship, the boats swinging clear and rockets, syren, and wireless calling aid from far and near.

Apparently the ship was fast settling by the head. A mine explosion had torn a widely gaping hole forrard, and her final plunge seemed only a matter of minutes away, but order prevailed. Boat after boat, crammed with men, got away into the darkening night, fading, with an echoing cheer, away from the pale ring of emergency lights which a solitary oil engine had kept going. It is a story told and retold in the war-time Press, perhaps with many gross exaggerations, but to those on deck, the wonderful discipline of our civilian army in the hazardous work of filling each boat in the choppy seas made a more than impressive experience. Many fractures and minor injuries occurred in that last jump for the crowded lifeboats, and it was amazing that not one life was lost in the process.

### Ashore.

A hospital training in no sense equips one for handling an open boat in the greater seas; somehow it is not easy to bring a small craft alongside a big merchantman, and still less so to do it in such a fashion that allows the human freight to spring easily to an inviting rope ladder swung overside. An exhausting process it was, and we will draw a veil across the record of many misguided efforts. Fortune was kind and made allowance perhaps, but suffice it to say that the half-clothed, chilled occupants of boat No. 16 viewed from another, and horizontal deck, the return of *T*——. to Cape Town. Urged by tugs and light warships, she crawled into harbour, foredecks awash, a far less dignified sight than on her previous and band-accompanied visit. Of the battalion, all were brought in. Boots, caps, tunics, gear of all kinds had gone astray, but not a man was lost. A truly nondescript collection of Britishers boarded the train for Wynberg, and with arrival at that delightful spot, we may let this story end. H.M. Transport *T*——. had never deserved our criticism. Her voyage and her misfortune were far from unique in days of world-wide horror, but her story strikes a note typical of the times and of Britain's world-wide war activities.

